# In Vitro Biodegradation Pattern of Collagen Matrices for Soft Tissue Augmentation

**DOI:** 10.3390/polym13162633

**Published:** 2021-08-07

**Authors:** Cristina Vallecillo, Manuel Toledano-Osorio, Marta Vallecillo-Rivas, Manuel Toledano, Raquel Osorio

**Affiliations:** 1 Faculty of Dentistry, Colegio Máximo de Cartuja s/n, University of Granada, 18071 Granada, Spain; cvallecillorivas@hotmail.com (C.V.); mvallecillo@correo.ugr.es (M.V.-R.); toledano@ugr.es (M.T.); rosorio@ugr.es (R.O.); 2 Medicina Clínica y Salud Pública PhD Programme, University of Granada, 18071 Granada, Spain

**Keywords:** keratinized tissue, mucosal thickness, soft tissue graft, collagen matrices

## Abstract

Collagen matrices have become a great alternative to the use of connective tissue grafts for soft tissue augmentation procedures. One of the main problems with these matrices is their volume instability and rapid degradation. This study has been designed with the objective of examining the degradation of three matrices over time. For this purpose, pieces of 10 × 10 mm^2^ of Fibro-Gide, Mucograft and Mucoderm were submitted to three different degradation tests—(1) hydrolytic degradation in phosphate buffer solution (PBS); (2) enzyme resistance, using a 0.25% porcine trypsin solution; and (3) bacterial collagenase resistance (*Clostridium histolyticum*)—over different immersion periods of up to 50 days. Weight measurements were performed with an analytic microbalance. Thickness was measured with a digital caliper. A stereomicroscope was used to obtain the matrices’ images. ANOVA and Student–Newman–Keuls tests were used for mean comparisons (*p* < 0.05), except when analyzing differences between time-points within the same matrix and solution, where pair-wise comparisons were applied (*p* < 0.001). Fibro-Gide attained the highest resistance to all degradation challenges. The bacterial collagenase solution was shown to constitute the most aggressive test as all matrices presented 100% degradation before 14 days of storage.

## 1. Introduction

The oral mucosa comprises both masticatory and alveolar mucosa. The nonattached alveolar mucosa is thin and is mostly composed of loosely affiliated collagen fibers while the attached mucosa is fixed, thick, keratinized, and is composed of well-organized, dense, collagen fibers [1]. An adequate amount of attached keratinized mucosa is essential for the maintenance of teeth and dental implants [1,2].

Notwithstanding the recognized value of a sufficient amount of keratinized mucosa, scientific evidence reports controversial data about the thickness and amount required. Previous findings have stated that a keratinized mucosa width of less than 2 mm more frequently exhibits clinical signs of inflammation, whereas sites showing ≥2 mm of keratinized mucosa remained healthy [3,4]. It was established that an adequate width of keratinized mucosa (≥2 mm) may be necessary in order to maintain periodontal health. These findings were later challenged by other authors who demonstrated that clinical signs of inflammation can be detected regardless of keratinized mucosa width [5,6]. As with teeth, the effect of an adequate width of a keratinized mucosa around dental implants on the stability of peri-implant tissues is still a matter of debate. However, it is clear that the lack of keratinized mucosa is not always associated with increased bone loss or peri-implant disease, but an adequate amount of it may be more favorable for the long-term maintenance of dental implants [1,7]. The presence of keratinized mucosa helps to preserve soft and hard tissues, favoring oral hygiene and preventing recession over time [1,7]. In addition, the absence of a sufficient width and thickness of periodontal or peri-implant soft tissues can compromise the aesthetics or function [8,9].

Soft tissue augmentation procedures have been proposed to treat mucogingival deformities and achieve both aesthetic and functional results, increasing the survival rates of teeth and dental implants [10]. Many surgical techniques have been performed to augment gingival tissue dimension. These procedures are categorized into two main groups: methods involved in keratinized mucosa generation and/or widening, and those causing increases in the volume/thickness of soft tissue [11]. Regardless of the technique used, autogenous connective tissue grafts are currently the gold standard [7]. This autogenous graft requires a donor area, increasing morbidity in terms of post-operative discomfort and procedure time [12]. Taking an autograft harvested from the palatal implies possible complications such as bleeding due to damage of the branches of the palatine artery, necrosis of the mucosa and hypo- or anaesthesia [13]. In order to avoid these complications related to the second surgical site, biomaterials are being used as replacements. Such alternative products can be broadly divided into different groups according to their origin: allogeneic, xenogeneic, and synthetic (alloplastic) materials [1]. Acellular dermal matrix allograft obtained from human cadaveric skin may be associated with ethical concerns and potential risk of disease transmission [14]. Xenogeneic porcine collagen matrices, processed to remove antigenic cellular components, have been described as an unlimited alternative to acellular dermal matrix and connective tissue grafts, with comparable results to autogenous soft tissue constructs [15,16]. These substitute biomaterials act as a three-dimensional scaffold to allow, at the same time, continuous production of new connective tissue and degradation of the original matrix [13,15]. The process causes the material to gradually be replaced by host tissues.

Unlike the membranes for Guided Bone Regeneration whose function is occlusive and space maintenance [17], for the purpose of soft tissue augmentation, a suitable material needs to fulfill two main criteria—stability of volume over time and favorable biological behavior—allowing modeling and remodeling processes [12]. Biodegradation and subsequent reorganization of the collagen matrix has important implications in terms of cell proliferation and organization with the creation of a suitable functioning tissue structure [18]. The rapid biodegradation by the enzymatic activity of periodontopathic bacteria, macrophages and polymorphonuclear leucocytes of native collagen is still a concern [13,19]. Furthermore, the degradation of collagen-based biomaterials is accentuated when the substitute is exposed to the oral environment [20]. The fiber strength and degradation rate can be influenced by combining collagen with other materials, by synthetically induced crosslinking, or by adding more thickness of collagen [21]. Various studies have been carried out to investigate the influence of membrane thickness on durability with the addition of a second membrane layer [21,22]. Although the rate of collagen degradation is similar in both configurations, the application of an additional layer results in a significantly higher amount of residual collagen [21]. This suggests that matrices with a higher collagen content and thickness should degrade more slowly. In addition, in order to decelerate resorption and, thus, prolong the barrier function of collagen matrices, several cross-linking methods, such as ultraviolet radiation, glutaraldehyde, diphenyl-phosphorylazide or hexamethylenediisocyanate, have been used [21,23]. It is commonly known that these processes increase the number of links between collagen molecules [24,25]. This procedure induces the formation of stable intra- or intermolecular chemical bonds, strengthens collagen matrices, and slows down enzymatic degradation [24]. The reinforcement of matrix materials through the cross-linking of collagen treated by different physical/chemical methods has been previously demonstrated [26,27,28]. However, these cross-linked collagen matrices are not usually employed clinically, because it has been shown that cross-linking using glutaraldehyde decreased biocompatibility, whereas enzymatic cross-linking reduced the tissue integration and biodegradation pattern [23]. In addition, increased cross-linking diminishes water absorption and increases membrane stiffness, which may affect clinical manageability [21].

Membranes that are commercially available for soft tissue augmentation have been clinically shown to report favorable results, including the recent stable volume collagen matrix with cross-linked collagen [7,29,30]. However, due to the frequency of exposure of the membranes to the oral environment during the healing period, it would be interesting to observe the behavior of these matrices against media that represent similar conditions as exposure to the oral environment. Collagen matrices with different cross-linked structures exhibit significant differences in biodegradation pattern, resorption time, and extent of inflammatory cell invasion [31]. Therefore, it can be assumed that the reaction behavior of matrices with and without collagen cross-linking to immersion media will be different. The exposed matrices are expected to be submitted to more aggressive environments than those in submerged healing. Several studies have assessed the degradation of guided bone regeneration membranes [23]; however there is no research that elucidates the potential in vitro degradability associated with the collagen matrix for soft tissue augmentation procedures. The aim of the present study was to evaluate the biodegradation pattern of three commercially available collagen matrices, one of them cross-linked and two without any cross-linking process, over different immersion periods up to 50 d. Qualitative and quantitative analyses of the collagen matrix degradation were performed. It was hypothesized that: *(i)* the three matrices for soft tissue augmentation do not show the same degradation kinetics over time; and *(ii)* the three matrices do not resist, in a similar manner, the different degradation tests (hydrolytic, bacterial collagenase and trypsin).

## 2. Materials and Methods

### 2.1. Matrices Description

Three experimental porcine collagen matrices, developed for soft tissue augmentation procedures, were tested. The matrices are commercially available and CE-certified for oral applications and are based on the same native dermal Type I and III collagen tissue. The evaluated matrices were: (i) Geistlich Fibro-Gide (Geistlich Pharma AG, Wolhusen, Switzerland); (ii) Geistlich Mucograft (Geistlich Pharma AG, Wolhusen, Switzerland); (iii) Mucoderm (Botiss biomaterials GmbH, Zossen, Germany). According to the manufacturer, Geistlich Fibro-Gide is a porous, resorbable and volume-stable matrix, made of reconstituted collagen, which undergoes smart chemical cross-linking. The matrix is provided in 6 mm thickness, and it can be applied in either a dry or wet state according to individual preference, but swelling upon wetting must be taken into account when determining final dimensions, as the matrix will gain approximately 25% in volume. Geistlich Mucograft is a fully resorbable collagen matrix made of porcine collagen without further cross-linking. As indicated by the provider, Geistlich Mucograft is approximately 2.5–5.0 mm thick with two structures: a compact collagen structure with a smooth texture that provides appropriate elastic properties, and a second structure consisting of a thick, porous collagen spongious scaffold. No prior hydration is required. Finally, Mucoderm is a resorbable matrix of 1.2 to 1.7 mm thickness, which is made of pure porcine collagen without any artificial/chemical cross-linking. The elastic properties of the matrix change with hydration, and the flexibility of the Mucoderm graft increases with hydration time, so a sufficiently long hydration, 5 to 20 min, is required before application.

Fibro-Gide has only one porous layer consisting of 60–96% (*w*/*w*) porcine collagen and 4–40% (*w*/*w*) elastin [32]. The biomaterial has 93% volume porosity, and cross-linked collagen displays a trabecular structure that forms large honeycomb-like interconnected pores, consisting of an amorphous, sheet-like matrix [30]. A study concluded that the sheet-like matrix was collagen, and the small fibers were elastin [33]. Mucograft is composed of a porcine collagen bilayer structure that presents two surfaces. The compact layer consists of condensed collagen fibers with occlusive cellular properties, allowing tissue adherence as a prerequisite for favorable wound healing. This layer is expected to protect against bacterial infiltration during open healing conditions and offers elastic properties to accommodate suturing. The second layer consists of a thick, porous, spongy collagen structure, which is placed next to the host tissues to facilitate organization of the blood clot, promoting both neoangiogenesis and tissue integration [5,34]. Mucoderm is a 3D collagen tissue matrix derived from porcine dermis containing collagen and elastin [35]. The collagen fibers are disposed as a loose mesh, forming an open-porous collagen structure that guides soft tissue cells and blood vessels [36]. The main characteristics of the matrices used in this biodegradation study are listed in Table 1.

### 2.2. Degradation Assays

The matrices were cut into pieces of the same size to obtain equal 10 × 10 mm^2^ samples from each matrix. Three specimens of each matrix type were employed for each test, and weight and thickness measurements were acquired for each specimen. An analytic scale (A&D-Instruments, Frankfurt, Germany) mounted on an anti-vibratory table was used for weight (W) measurements, with an accuracy of 0.0001 g. Thickness (Th) was measured at three random positions by means of a digital caliper (Mitutoyo 293-561, Tokyo, Japan). Three different degradation tests were performed:

(1) Hydrolytic degradation test: immersion of specimens in phosphate buffer solution (PBS) at 37 °C [37].
(2) Enzyme resistance test: samples were immersed in a 0.25% porcine trypsin solution (Sigma-Aldrich, St. Louis, MO, USA) at 37 °C [31].
(3) Bacterial collagenase resistance test: collagenase solution from *Clostridium histolyticum* bacteria Type V (Sigma-Aldrich, St Louis, MO, USA) was used. This solution contained a mixture of several different enzymes including collagenase, which act together to break down tissue. This preparation contains collagenase, non-specific proteases, clostripain, neutral protease, and aminopeptidase activities. Specific activity is ≥125 CDU/mg solid. A collagenase concentration of 2 IU/mL in 50 mM Tris HCl (pH = 7.4), containing 10 mM CaCl_2_, was used [19,24]. After each 48 h, degradation solutions were carefully removed through suction and renewed [38].

### 2.3. Statistical Analysis

Normal distribution of data was probed using the Kolmogorov–Smirnov (KS) test (*p* > 0.05) Multiple ANOVA models were used to assess the influence of the independent variables (degradation solution, type of matrix and immersion time) on the dependent variables (weight and thickness). Analyses of interactions were also performed. ANOVA and Student–Newman–Keuls post-hoc comparisons were performed to determine differences between materials and degradation solutions. To permit for these comparisons, the weight and thickness variables were converted to percentages of variation with respect to the initial measurement, using the following Equation (1):

Percentage of loss = [(X_0_ − X_t_)/X_0_] × 100, (1)

where X_0_ is the initial weight or thickness of specimens, and X_t_ is the specimen’s weight or thickness at each time point (t).

Pairwise comparisons were performed to ascertain the differences between immersion time-points within the same matrix and solution experimental group. Statistical significance was always considered at *p* < 0.05 except for pairwise comparisons, where a Bonferroni’s correction was applied and *p* < 0.001 was set. Statistical analysis was performed using the SPSS 25.0 (SPSS Inc., Chicago, IL, USA) software package.

### 2.4. Light Microscopy Analysis

Before starting the matrices’ immersion and at the end of the storage period 7 d, specimens were observed under an Olympus SZ-60 stereomicroscope (Olympus, Tokyo, Japan) for microstructural analysis. Images were taken at 60× and 120× magnifications.

## 3. Results

**Figure 1:**
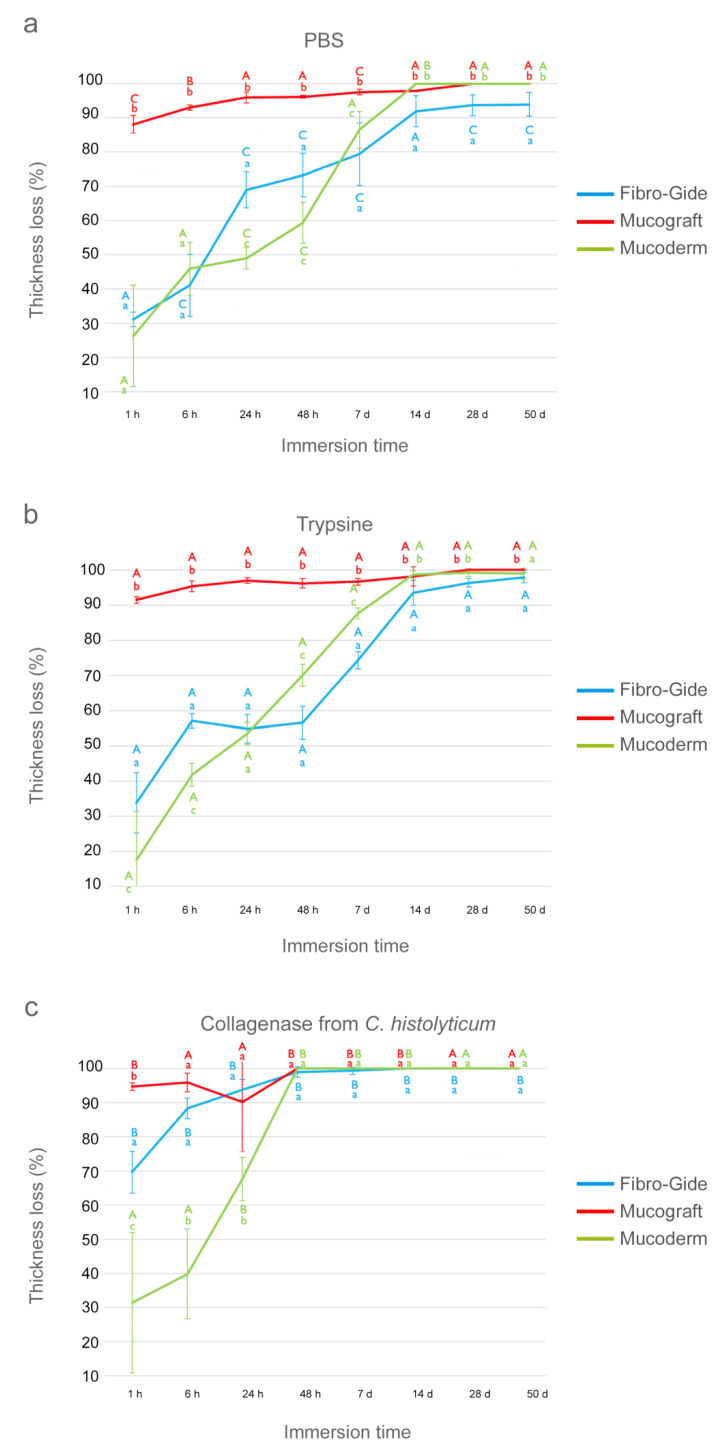
Percentage of thickness loss of the three matrices studied after immersion periods of up to 50 days in: (**a**) PBS, (**b**) trypsin and (**c**) *C. histolyticum* collagenase. Values shown are mean and standard deviation. Statistical significance is indicated with lower-case letters for differences between matrices over the same degradation test and with capital letters for differences between degradation tests within the same membrane. Student–Newman–Keuls tests were performed for multiple comparisons (*p* < 0.05).

The thickness (Th) values in mm of the three matrices studied (Fibro-Gide, Mucograft and Mucoderm), subjected to different solutions (PBS, trypsin and *C. histolyticum* collagenase) during different submersion time periods (1 h, 6 h, 24 h, 48 h, 7 d, 14 d, 28 d, 50 d), are shown in Table 2. The thickness (Th) loss values of the matrices, expressed in percentage, across different degradation tests and times, are represented in Figure 1.

### 3.1. Thickness Evaluation after PBS Degradation Assay

At the 1 h time-point, Mucograft attained higher loss of thickness, close to 90%, while Fibro-Gide and Mucoderm performed similarly with losses of Th of approximately 30% (Figure 1a). After 24 h of storage, Mucograft reached the highest and Mucoderm the lowest Th percentage values; the trend was as follows: Mucograft > Fibro-Gide > Mucoderm (Figure 1a). After 7 d of immersion, Fibro-Gide had the lowest and Mucograft the highest loss of Th, and Mucoderm reached intermediate performance between both. After 14 d of storage, the highest loss of Th was attained by Mucoderm, which totally degraded. Fibro-Gide showed the lowest loss of Th values. The trend was as follows: Mucoderm > Mucograft > Fibro-Gide (Figure 1a). At 28 and 50 d, only Fibro-Gide remained, and Mucograft did not exceed 28 days of immersion.

In general terms, the three matrices had significant ascending loss of thickness according to the different time-points of the study when compared to the initial values, except for Mucoderm, which attained significant differences after 6 h of storage (Table 2). Mucoderm was completely degraded after the 14 d immersion period (Figure 1a). Mucograft was fully degraded during a period of 28 d of immersion, and Fibro-Gide, with very low values of thickness, overcame 50 d of storage (Figure 1a).

### 3.2. Thickness Evaluation after Trypsin Degradation Assay

After 1 h of storage, Mucograft suffered the greatest loss of Th percentage followed by Fibro-Gide and finally Mucoderm. At the 6 h time-point, the matrices reduced their percentage Th as follows: Mucograft > Fibro-Gide > Mucoderm (Figure 1b). After 24 h of storage, Mucoderm and Fibro-Gide performed similarly with lower losses of thickness than Mucograft. After 14 d, the trend changed as follows: Mucoderm ≥ Mucograft > Fibro-Gide. After 50 d of immersion, all matrices remained, although with high losses of Th percentage following this trend: Fibro-Gide > Mucograft > Mucoderm (Figure 1b).

In general terms, within this degradation test, all matrices experienced significant changes in their thickness throughout the different time-points, except for Mucoderm, which attained significant differences after 6 h of storage (Table 2). Although no matrix was completely degraded after 50 days of storage, the degradation values were high, and the percentage of thickness loss was above 95% (Figure 1b).

### 3.3. Thickness Evaluation after C. histolyticum Collagenase Degradation Assay

At the 1 h time-point, the three matrices suffered a marked loss of their thickness, which was greater in Mucograft and lower in Mucoderm (Figure 1c). After 24 h of storage, Fibro-Gide attained the highest loss of Th percentage values, and the trend was as follows: Fibro-Gide > Mucograft > Mucoderm (Figure 1c). At the 48 h time-point, Mucograft and Mucoderm completely degraded; the loss of Th was complete, and only Fibro-Gide exceeded this period, totally degrading after 7 days.

In general terms, the *C. histolyticum* collagenase degradation assay was the most aggressive with the matrices studied (Table 2). All matrices experienced a significant reduction in their thickness values over time, when all time-points were compared with the initial time, except Mucoderm, which attained significant differences after 6 h of storage (Table 2). None of them completed all study periods. Mucograft and Mucoderm were completely degraded during 48 h of immersion, and Fibro-Gide managed to exceed 7 days but did not reach 14 days (Figure 1c).

**Figure 2:**
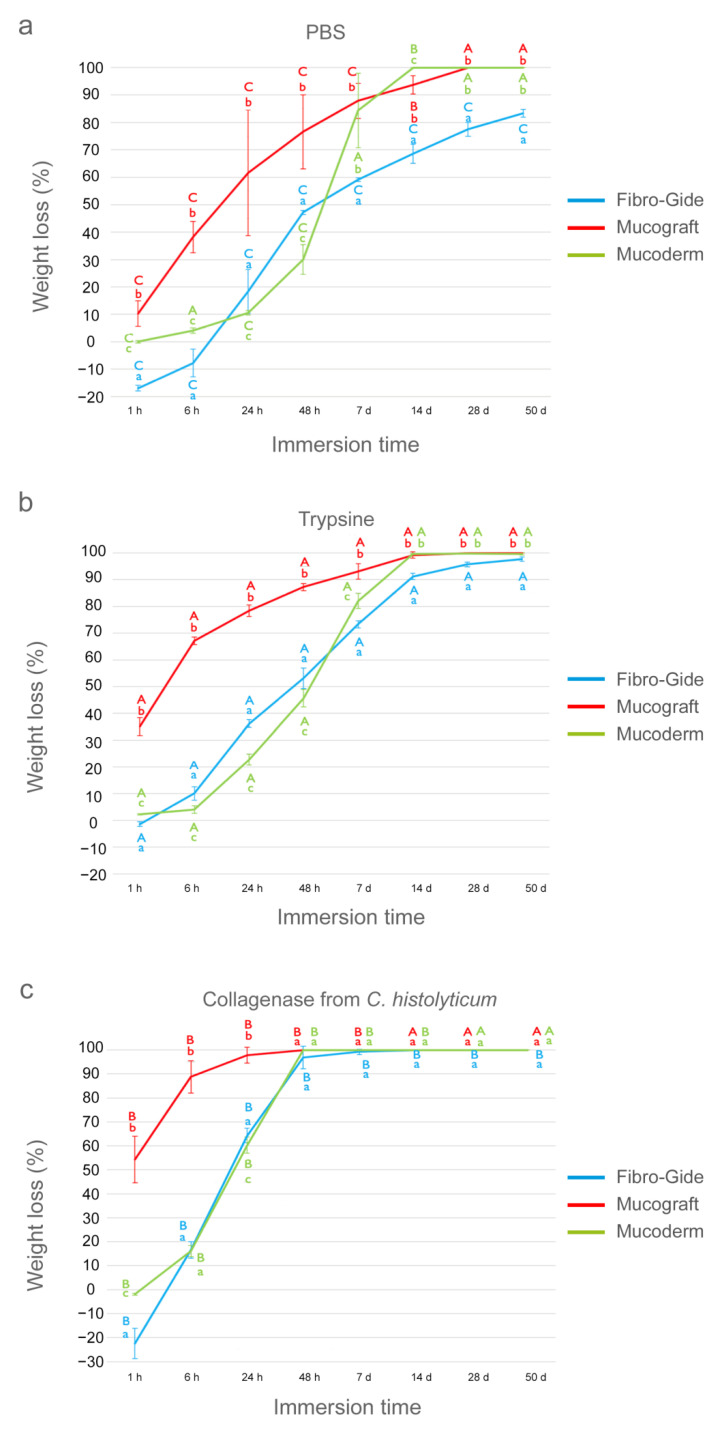
Percentage of weight loss in the three tested matrices after the different immersion periods of up to 50 days in: (**a**) PBS, (**b**) trypsin and (**c**) *C. histolyticum* collagenase. Values are shown as mean and standard deviation. Statistical significance is shown with lower-case letters for differences between matrices over the same degradation test, and with capital letters for differences between degradation tests within the same membrane. Student–Newman–Keuls tests were performed for multiple comparisons (*p* < 0.05).

The weight (W) values in g of the three matrices studied (Fibro-Gide, Mucograft and Mucoderm) subjected to different solutions (PBS, trypsin and *C. histolyticum* collagenase) during different submersion time periods are presented in Table 3. The weight (W) loss values, expressed in percentage, over different degradation tests and times, are shown in Figure 2.

### 3.4. Weight Evaluation after PBS Degradation Assay

At the 1 h time-point, Fibro-Gide achieved a weight gain of almost 20%, while Mucograft attained the highest loss of W values. Mucoderm did not suffer any change in its weight (Figure 2a). The weight gain values that were revealed were due to water absorption that likely occurred during the first matrix immersion. After 24 h of storage, the three matrices experienced loss of weight, and the trend was as follows: Mucograft > Fibro-Gide > Mucoderm. After 7 d of immersion, Mucoderm showed a marked change in percentage W values, which were similar to those of Mucograft. Fibro-Gide attained the lowest loss of weight (Figure 2a). At the 28 d time-point, Mucograft and Mucoderm were completely degraded as the loss of W was complete for both of them. Fibro-Gide exceeded 50 d of storage with a weight loss of 70% (Figure 2a).

In general terms, the three matrices underwent significant changes in their weight when compared with the initial weight; these changes were progressive for Fibro-Gide and Mucograft, while Mucoderm suffered a gradual reduction in weight until 7 days, in which the degradation became marked and almost complete (Table 3). Mucograft totally degraded at 28 d of immersion and Fibro-Gide exceeded the 50 d time-point (Figure 2a).

### 3.5. Weight Evaluation after Trypsin Degradation Assay

At the 1 h time-point, Mucograft attained the highest loss of W, and Fibro-Gide and Mucoderm hardly experienced changes (Figure 2b). After 14 h of storage, Mucograft showed the highest and Mucoderm the lowest loss of percentage weight, with the following trend: Mucograft > Fibro-Gide > Mucoderm. After 7 d of immersion, this trend changed as follows: Mucograft > Mucoderm > Fibro-Gide (Figure 2b). From 14 d to 50 d of storage, Fibro-Gide showed the lowest loss of percentage W values, and both Mucograft and Mucoderm performed similarly (Figure 2b).

In general terms, after completing the different study periods, the three matrices suffered significant weight loss over time in this degradation assay (Table 3). After 50 days, the highest weight values were for Fibro-Gide and the lowest for Mucoderm. The final trend was Fibro-Gide > Mucograft > Mucoderm (Figure 1b).

### 3.6. Weight Evaluation after C. histolyticum Collagenase Degradation Assay

At 1 h time-point, Mucograft was the only matrix that experienced loss of W (Figure 2c). After 6 h of storage, Mucograft attained the highest loss weight. Mucoderm and Fibro-Gide suffered similar losses of W, which were close to 15%. At the 48 h time-point, both the Mucograft and Mucoderm matrices completely degraded. At the 7 d time-point, a complete degradation was attained for all of them (Figure 2c).

In general terms, this solution aggressively affects matrices, obtaining the lowest weight values over periods studied (Table 3). None of them overcame 50 d of storage. Mucograft and Mucoderm exceeded up to 24 h of immersion, degrading completely during 48 h in the presence of this test solution (Figure 1c). Fibro-Gide was partially degraded after 7 d and disappeared after 14 d of immersion.

### 3.7. Matrices Morphological Analysis

**Figure 3:**
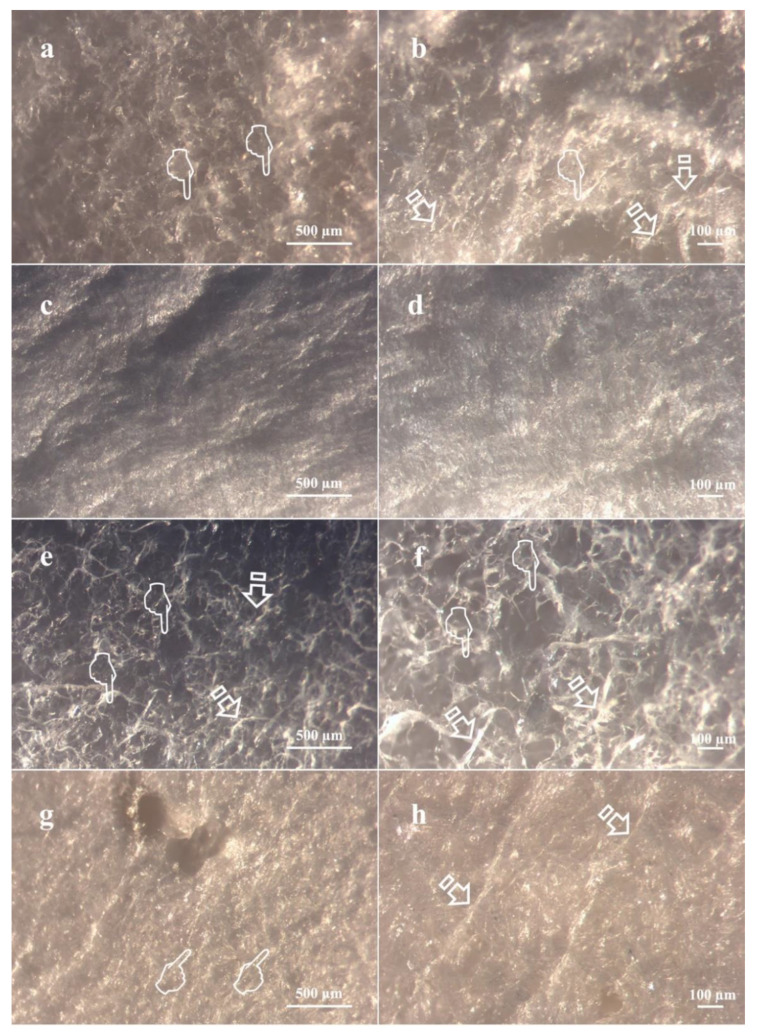
Light micrographs taken from matrices before immersion. (**a**) Fibro-Gide surface at 60× magnification. (**b**) Fibro-Gide at 120× magnification. (**c**) Compact layer Mucograft surface at 60× magnification. (**d**) Compact layer Mucograft surface at 120× magnification. (**e**) Spongious layer Mucograft surface at 60× magnification. (**f**) Spongious layer Mucograft surface at 120× magnification. (**g**) Mucoderm surface at 60× magnification. (**h**) Mucoderm surface at 120× magnification. Scale bar is 500 µm in (**a**,**c**,**e**,**g**), and 100 µm in (**b**,**d**,**f**,**h**). Pointers indicated the presence of different sized pores. Arrows show collagen fibers distributed over matrices’ surfaces.

**Figure 4:**
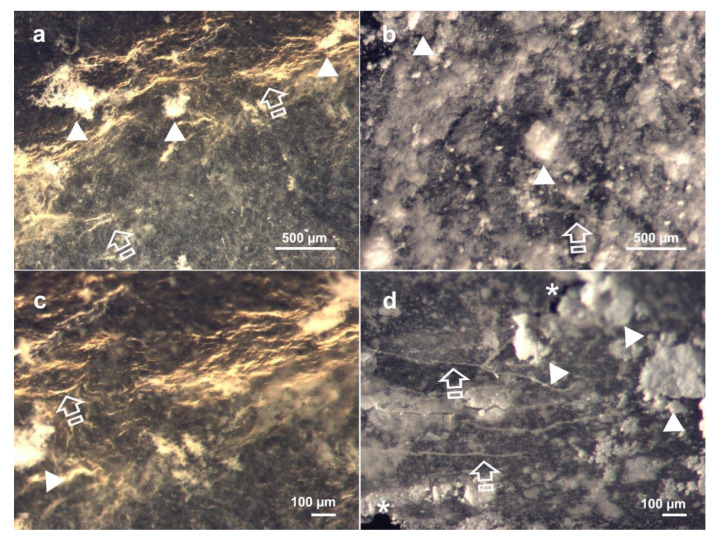
Light micrographs taken from matrices after PBS degradation assay after 7 days of storage. (**a**) Fibro-Gide surface at 60× magnification. (**b**) Mucograft surface at 60× magnification. (**c**) Fibro-Gide at 120× magnification. (**d**) Mucograft at 120× magnification. Scale bar is 500 µm in (**a**,**c**); and 100 µm in (**b**,**d**). Different layers of collagen fibers (arrows) caused by degradation with some mineral deposits (arrow heads) on its surface. The lack of integrity of the matrices corresponds with structural defects (asterisks).

**Figure 5:**
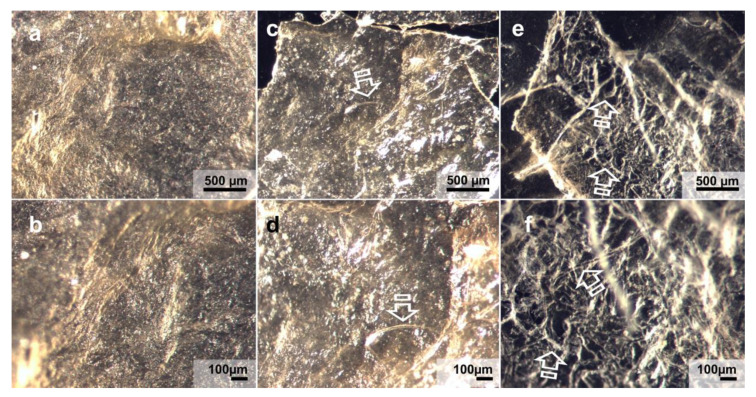
Light micrographs taken from matrices after Trypsin degradation assay after 7 days of storage. (**a**) Fibro-Gide surface at 60× magnification. (**b**) Fibro-Gide surface at 120× magnification. (**c**) Mucograft surface at 60× magnification. (**d**) Mucograft surface at 120× magnification. (**e**) Mucoderm surface at 60× magnification. (**f**) Mucoderm surface at 120× magnification. Scale bar is 500 µm in (**a**–**c**), and 100 µm in (**d**–**f**). Disorganized fiber structure caused by partial degradation with rippled collagen fibrils (arrows) is observed.

Figure 3 displays light micrographs taken from the three matrices before they were submitted to immersion into the degradation solutions. Fibro-Gide showed a rough surface (Figure 3a,b), with exposed collagen shaping different sized pores, ranging from 100 to 250 µm. Mucograft had two differentiated surfaces; the outer substrate was smooth, homogeneous and non-permeable (Figure 3c,d), while the inner one displayed numerous pores (Figure 3e,f). Lastly, Mucoderm (Figure 3g,h) exhibited a porous surface with noticeable parallel collagen fibrils. The pores of this membrane were more homogeneous in shape and size, between 80 µm and 100 µm. Figure 4 and Figure 5 show light micrographs taken from the matrices after 7 days of storage in the degradation tests, PBS (Figure 4) and Trypsin (Figure 5). Due to the severe resorption of the matrices, at this time-point in the *C. histolyticum* collagenase degradation assay, no images of microstructural alteration could be obtained with this solution for any matrix. The reaction of Mucoderm to the PBS degradation assay after 72 h could also not be evaluated with light micrographs.

Figure 4 shows how the PBS immersion test altered the structure of the matrices with an irregular exposure of the different layers of collagen. The breakdown of collagen at the Fibro-Gide membrane made it impossible to distinguish the initially described pores (Figure 4a). Some mineral deposits could be seen on the matrices’ surface (Figure 4a). After 7 days in PBS, different Mucograft surfaces turned out to be indistinguishable. The previously described porous structure also disappeared, and both surfaces were equally smooth (Figure 4b,d). Some collagen fibrils remained intact; however, there were some points in which lack of integrity of the matrices could be noticed (Figure 4d).

After trypsin immersion, the three matrices showed a disorganized fiber structure, caused by the partial collagen degradation generated by the peptidase. The most affected membrane was Mucoderm, which showed rippled collagen fibrils with disorganized and variable orientation (Figure 5e,f). This disorganized collagen fibrils could also be observed, but to a lesser degree, in Mucograft (Figure 5c,d). From a microscopic point of view, Fibro-Gide was the matrix that remained less disrupted after the trypsin challenge (Figure 5a,b). It had lost its initial clear porous structure, although it still exhibited a structured collagen net.

## 4. Discussion

The obtained data showed that the three matrices were progressively degraded throughout the immersion periods and did not resist the degradation tests equally. Fibro-Gide has been the matrix that better resisted degradation tests measured in terms of weight and thickness (Figure 1 and Figure 2).

The greater resistance to degradation presented by Fibro-Gide could be explained by the difference in the composition with respect to the other tested matrices. Although Mucograft, Mucoderm and Fibro-Gide largely consist of the same material, native porcine collagen type I and III, Fibro-Gide is smart-linked, a unique form of cross-linking, which provides elasticity, strength and volume stability. Cross-linking of collagen fibers by physical, thermal or chemical methods has demonstrated to increase resistance to degradation [13] if compared to the native state of collagen. In this study, Fibro-Gide reached the highest values of weight and thickness in the final stages (Figure 1 and Figure 2), showing better resistance than the native type I and III collagen matrices, Mucograft and Mucoderm, to any degradation medium. This may be due to the cross-linking of Fibro-Gide. It was the only matrix that after 7 days of trypsin challenge preserved a structured collagen network, when analyzed by light micrographs (Figure 5). In the trypsin test, Fibro-Gide reached a degradation close to 98% of its weight and thickness at 50 days, while Mucograft and Mucoderm degradation reached 98% at only 7 days of storage (Figure 1 and Figure 2). In the PBS test, only Fibro-Gide exceeded 50 days of immersion with a degradation of 94%, while Mucograft was totally degraded at 28 days and Mucoderm at 14 days of storage (Figure 1 and Figure 2). After the bacterial collagenase resistance test, all matrices suffered an accelerated degradation, which was from 1 h to 14 days for Fibro-Gide, from 1 h to 48 h for Mucograft, and from 1 to 24 h for Mucoderm (Figure 1 and Figure 2). These differences in the degradation resistance between matrices with cross-linked and non-cross-linked collagen fibers were also found by other authors. Rothamel et al. [13] evaluated the implantation of different types of matrices in subcutaneous rat models to observe biodegradation, vascularization and tissue integration. As in the present study, the aforementioned research found a more accelerated degradation of the non-cross-linked matrix compared to those that presented collagen cross-linking. However, its results are only attributable to submerged healing, while in our study, different media of immersion represented different degradation scenarios. This allows us to affirm that differences between matrices with cross-linked and non-cross-linked collagen were maintained regardless of the exposure medium. Sela et al. [19] also attributed a greater resistance to degradation with the use of cross-linked membranes. They compared Biomend, a cross-linked membrane, with Biogide, a non-cross-linked membrane, in the presence of different oral pathogens, but only for 16 h, which is insufficient to determine membranes’ long-term degradation behavior. In general terms, it can be confirmed that the presence of cross-linked collagen fibers in the composition of a matrix causes greater stability and resistance against degradation tests [13,24,39].

Predicting matrices’ behavior and their degradation in different scenarios is essential for the collagen matrices used in soft tissue augmentation. It is indispensable that the collagen matrices to be incorporated and accepted in the recipient tissue, and present adequate volumetric stability, in order to allow enough time for cell and blood vessel infiltration [40]. Migration, activation and proliferation of endothelial cells from existing vessels is required [40]. Eppley et al. [41] demonstrated in an in vivo animal model that a single-layer acellular human dermis membrane was revascularized quickly, with this process being completed by the second postoperative week. It was considered that 14 days was sufficient time for the cell invasion and revascularization. However, it has been demonstrated that the integration of collagen membranes of human and porcine origin is different, despite the similarity in morphology and behavior of porcine and human skin [40,42]. In the case of porcine collagen matrices, it has been shown that this time is significantly longer, and the vascularization of the central zone of the matrix occurs between 30 days and 60 days [34,43,44]. This is the reason why the matrix must remain intact for at least 4 weeks in order to allow the incorporation and regeneration of oral tissues [24]. Rothamel et al. [13] stated that, after four weeks of implantation, matrices were vascularized and the degradation was practically completed. The studied matrices, including Mucoderm, were organized in new connective tissue at 4 weeks and they also found remains of matrices even after 12 weeks [13]. The three matrices were able to overcome the submersion in trypsin after 28 days of storage, demonstrating survival in the enzyme resistance test. Only Fibro-Gide was able to withstand 28 days of storage in PBS, but none of the three matrices exceeded this experimental period in *C. histolyticum*. From 0 h to 1 h of immersion, the weight values increased for Fibro-Gide. This could be explained in terms of water absorption that likely occurs during the first immersion. Although the adsorbed water is probed to be similar for the wetting and drying processes, indicating that the collagen is at a close equilibrium of water adsorption/desorption [45], the initial water absorption is integrated into collagen by different states, i.e., bound and free water. Bound water, where water molecules form bridges between neighboring polypeptide chains, acts as a receptor for CH–O hydrogen bonds [46]. Free water is water that is fixed by one hydrogen bond between polypeptide chains or fixed in the hole zones at the end of the polypeptide chain [47]. Bound water needs specific methods to be removed from the collagen once it has been incorporated. Therefore, thicker matrices with higher collagen content absorb more water from the medium, with a part of it being incorporated into their structure as bound water [43]. In conclusion, our results show that not all matrices resisted 28 days of storage in all immersion media. In contrast, Owens and Yukna [48], who studied the animal implantation of collagen membranes for guide bone regeneration, established that the degradation was moderate to severe between 4 and 8 weeks; it should be stressed that membranes are still present after 8 weeks. The higher permanence of the membranes in the aforementioned studies [13,48] may be due, not only to the different compositions, but also to the fact that, in both cases, the membranes are placed at subcutaneous pockets. In our study, three in vitro tests have been used and probably represented more aggressive conditions than submerged healing. *C. histolyticum* has been the most aggressive test against matrices. The degradation by bacterial collagenase in vitro could predict the behavior of the matrix within the worst scenario in the case of wound dehiscence [23]. In addition, the enzyme resistance test with trypsin and the hydrolytic degradation test with PBS also served to evaluate the matrices’ behavior when faced with medium oral exposure. Differently from Fibro-Gide, Mucograft and Mucoderm could be exposed to the oral cavity (Table 1). Therefore, a greater resistance to degradation than Fibro-Gide was expected. Mucograft, a bilayered collagen matrix, presents two layers: a smooth, occlusive and compact layer of dense collagen that allows possible open healing [49], and another porous layer to promote cell adhesion. Exposure of Mucoderm, according to recommendations, should always be avoided when used for recession coverage, and open healing is feasible only in the case of a vestibuloplasty [50].

Several studies have demonstrated the affinity and adhesion of bacteria to collagen [51,52,53]. Bacterial adhesion to collagen plays critical roles in the pathogenesis and persistence of infections caused by numerous bacteria [52]. Each collagen molecule is formed through the interactions of three protein polypeptides known as α-strands and recognized as a triple helix structure [51]. Many bacteria have evolved to produce collagen adhesins to interact with this group of proteins [52], which favor the binding to collagen. This suggests that in the presence of bacteria, such as those of the genera *Staphylococcus*, *Streptococcus*, *Enterococcus*, and *Bacillus*, an immediate contamination of the collagen matrix would occur, which implies its rapid degradation, as shown with the *C. histolyticum* test. Collagen cross-linking has been shown to strengthen the matrices against collagenase degradation for up to 7 days. However, this time is considered insufficient to achieve a complete conversion and regeneration of the soft oral tissue [24]. Therefore, doping of the matrices with an antibacterial agent should be considered in order to partially overcome the problem of rapid degradation, which is caused by bacterial contamination in those cases in which membrane exposure may occur.

This study suggests that the presence of cross-linked collagen fibers in the matrix represents the most important aspect in terms of increasing resistance to degradation. At this point, a balance needs to be achieved between the increasing of resistance to degradation and the avoidance of the drawbacks of many of the usually employed crosslinking methods, which are frequently related to severe inflammatory responses [13,49]. The observed morphological changes of collagen matrixes after degradation mainly include the disarrangement of the collagen web and the widening of the interfibrillar spaces, which are consistent with previously described alterations found after scanning and transmission electron microscopy analyses of water degradation of human collagen [54]. Further research is planned in order to ascertain whether these changes in collagen fibrils’ nanostructure are related to the mechanical properties of collagen membranes during the degradation process. The great contribution of bacterial collagenase in membranes’ degradation has also been shown, even for matrices composed of cross-linked collagen fibers. However, it should be considered that in vitro results can only be extrapolated to the clinical the scenario with certain limitations. The outcomes of this study support future research related to the addition of antibacterial agents to the matrices, with the aim of protecting them against bacterial colonization.

## 5. Conclusions

After the in vitro evaluation of the three porcine collagen matrices, it can be stated that the matrices do not have similar resistance in the different degradation tests. Fibro-Gide, a cross-linked collagen matrix, has shown the highest resistance to the tested degradation challenges, under in vitro conditions, demonstrating the efficacy of the cross-linking methods. The bacterial collagenase solution constituted the most aggressive test, as all matrices presented 100% degradation before 14 d. Pores larger than 100 µm appeared during the degradation processes of all tested membranes.

## Figures and Tables

**Table 1:** Main characteristics of collagen-based matrices included in the study.

Matrix	Collagen	Origin	Cross-Link	Structure	Thickness (mm)	Exposed to the Oral Environment
Fibro-Gide	Type I and III	Porcine	Yes	3D matrix	6	No Submerged healing
Mucograft	Type I and III	Porcine	No	2 layers: compact and spongious scaffold	2.5–5.0	No/Yes Submerged healing recommended
Mucoderm	Type I and III	Porcine	No	1 layer	1.2–1.7	Yes Open healing is feasible in the case of a vestibuloplasty.

**Table 2:** (**a**) Thickness values (mm) of the three experimental matrices (Fibro-Gide, Mucograft and Mucoderm) after different immersion periods ranging from 1 h to 50 days in the degradation tests; PBS (hydrolytic degradation test), *C. histolyticum* collagenase (bacterial collagenase resistance test) and trypsin (enzyme resistance test). Values are presented as means and standard deviations. (**b**) *p* values obtained after pairwise comparisons between matrices’ thicknesses after different immersion times and the initial thickness (thickness at t0: Th0). Statistical significance was considered at *p* ≤ 0.001.

(a)									
	Fibro-Gide			Mucograft			Mucoderm		
	Trypsin	*C. histolyticum*	PBS	Trypsin	*C. histolyticum*	PBS	Trypsin	*C. histolyticum*	PBS
t0	5.78 (0.19)	6.32 (0.12)	5.68 (0.22)	3.93 (1.58)	3.55 (0.74)	4.12 (0.43)	1.39 (0.08)	1.28 (0.18)	1.22 (0.16)
1 h	3.84 (0.59)	1.92 (0.35)	3.91 (0.17)	1.58 (1.99)	0.18 (0.01)	0.49 (0.12)	1.15 (0.23)	0.84 (0.12)	0.88 (0.1)
6 h	2.48 (0.07)	0.74 (0.18)	3.34 (0.48)	0.93 (1.19)	0.13 (0.07)	0.29 (0.059)	0.81 (0.07)	0.75 (0.05)	0.67 (0.19)
24 h	2.6 (0.16)	0.39 (0.19)	1.77 (0.35)	0.86 (1.15)	0.12 (0.08)	0.17 (0.07)	0.65 (0.05)	0.4 (0.02)	0.62 (0.06)
48 h	2.51 (0.22)	0.063 (0.095)	1.52 (0.39)	0.81 (1.08)	0 (0)	0.16 (0.02)	0.42 (0.05)	0 (0)	0.49 (0.05)
7 d	1.48 (0.12)	0.043 (0.065)	1.18 (0.56)	0.55 (0.69)	0 (0)	0.099 (0.027)	0.17 (0.02)	0 (0)	0.16 (0.06)
14 d	0.37 (0.19)	0 (0)	0.47 (0.27)	0.11 (0.09)	0 (0)	0.09 (0.008)	0.02 (0.01)	0 (0)	0 (0)
28 d	0.21 (0.06)	0 (0)	0.36 (0.18)	0.053 (0.08)	0 (0)	0 (0)	0.01 (0.02)	0 (0)	0 (0)
50 d	0.12 (0.08)	0 (0)	0.35 (0.2)	0.016 (0.02)	0 (0)	0 (0)	0.01 (0.02)	0 (0)	0 (0)
**(b)**									
Th_0_–Th_1h_	<0.001	<0.001	<0.001	<0.001	<0.001	<0.001	0.006	0.002	0.002
Th_0_–Th_6h_	<0.001	<0.001	<0.001	<0.001	<0.001	<0.001	<0.001	<0.001	<0.001
Th_0_–Th_24h_	<0.001	<0.001	<0.001	<0.001	<0.001	<0.001	<0.001	<0.001	<0.001
Th_0_–Th_48h_	<0.001	<0.001	<0.001	<0.001	<0.001	<0.001	<0.001	<0.001	<0.001
Th_0_–Th_7d_	<0.001	<0.001	<0.001	<0.001	<0.001	<0.001	<0.001	<0.001	<0.001
Th_0_–Th_14d_	<0.001	<0.001	<0.001	<0.001	<0.001	<0.001	<0.001	<0.001	<0.001
Th_0_–Th_28d_	<0.001	<0.001	<0.001	<0.001	<0.001	<0.001	<0.001	<0.001	<0.001
Th_0_–Th_50d_	<0.001	<0.001	<0.001	<0.001	<0.001	<0.001	<0.001	<0.001	<0.001

**Table 3:** (**a**) Weight values (µg) of the three experimental matrices (Fibro-Gide, Mucograft and Mucoderm) after different immersion periods ranging from 1 h to 50 days in the different degradation tests; PBS (hydrolytic degradation test), *C. histolyticum* collagenase (resistance test to bacterial collagenase) and trypsin (Enzyme resistance test). Values are presented as means and standard deviations. (**b**) *p* values obtained after pairwise comparisons between matrices’ thicknesses after different immersion time-points and the initial weight (weight at t0: W0). Statistical significance was considered at *p* ≤ 0.001.

(a)									
		Fibro-Gide			Mucograft			Mucoderm	
	Trypsin	*C. histolyticum*	PBS	Trypsin	*C. histolyticum*	PBS	Trypsin	*C. histolyticum*	PBS
t0	43.5 (1.38)	49.54 (1.97)	45.55 (2.79)	31.94 (9.63)	26.97 (5.33)	31.68 (5.02)	56.34 (2.13)	57.4 (3.8)	41.63 (1.67)
1 h	44.11 (1.77)	60.62 (2.93)	53.31 (3.62)	26.63 (14.24)	12.64 (4.42)	28.53 (5.28)	55.09 (2.09)	58.51 (3.77)	42.1 (1.59)
6 h	39.15 (2.28)	41.31 (2.48)	48.99 (1.78)	19.32 (16.18)	3 (1.78)	19.49 (3.08)	54.09 (2.53)	48.16 (3.75)	39.95 (1.86)
24 h	27.78 (1.26)	17.61 (0.86)	37 (2.51)	13.59 (11.84)	0.57 (0.86)	12.07 (7.67)	43.51 (1.7)	22.8 (2.75)	37.23 (1.36)
48 h	20.35 (1.06)	1.49 (2.24)	24.03 (1.24)	8.9 (8.5)	0 (0)	7.46 (4.77)	30.64 (2.36)	0 (0)	29.14 (2.63)
7 d	11.61 (0.56)	0.35 (0.53)	18.64 (1.41)	5.14 (5.38)	0 (0)	3.91 (2.45)	10.07 (1.19)	0 (0)	6.48 (5.77)
14 d	3.8 (0.39)	0 (0)	14.39 (2.41)	1.47 (1.75)	0 (0)	1.97 (1.09)	0.21 (0.16)	0 (0)	0 (0)
28 d	1.85 (0.32)	0 (0)	10.31 (1.71)	0.52 (0.77)	0 (0)	0 (0)	0.08 (0.13)	0 (0)	0 (0)
50 d	0.99 (0.3)	0 (0)	7.62 (1.06)	0.26 (0.39)	0 (0)	0 (0)	0.16 (0.25)	0 (0)	0 (0)
**(b)**									
W_0_–W_1h_	0.002	<0.001	<0.001	0.009	<0.001	<0.001	<0.001	<0.001	<0.001
W_0_–W_6h_	<0.001	<0.001	0.001	<0.001	0.001	<0.001	<0.001	<0.001	<0.001
W_0_–W_24h_	<0.001	<0.001	<0.001	<0.001	<0.001	<0.001	<0.001	<0.001	<0.001
W_0_–W_48h_	<0.001	<0.001	<0.001	<0.001	<0.001	<0.001	<0.001	<0.001	<0.001
W_0_–W_7d_	<0.001	<0.001	<0.001	<0.001	<0.001	<0.001	<0.001	<0.001	<0.001
W_0_–W_14d_	<0.001	<0.001	<0.001	<0.001	<0.001	<0.001	<0.001	<0.001	<0.001
W_0_–W_28d_	<0.001	<0.001	<0.001	<0.001	<0.001	<0.001	<0.001	<0.001	<0.001
W_0_–W_50d_	<0.001	<0.001	<0.001	<0.001	<0.001	<0.001	<0.001	<0.001	<0.001

## Data Availability

The data presented in this study are available on request from the corresponding author.
